# Ultrasound-Assisted Aqueous Two-Phase System for Extraction and Enrichment of *Zanthoxylum armatum* Lignans

**DOI:** 10.3390/molecules200815273

**Published:** 2015-08-20

**Authors:** Tao Guo, Dan Su, Yan Huang, Ya Wang, Yong-Hui Li

**Affiliations:** 1 School of Life Science and Engineering, Lanzhou University of Technology, Lanzhou 730050, China; E-Mails: gt010010@sina.com (T.G.); sudan58138@126.com (D.S.); hy123-0@163.com (Y.H.); wangya502@163.com (Y.W.); 2 Hainan Provincial Key Laboratory of R & D of Tropical Plants, Hainan Medical University, Haikou 571101, China

**Keywords:** *Zanthoxylum armatum*, lignans, aqueous two-phase system, ultrasonic-assisted extraction, artificial neural network, response surface methodology

## Abstract

In the study, an aqueous two phase system (ATPS) coupled with ultrasound was employed to extract lignans from *Zanthoxylum armatum*. Three standard lignans, namely (−)-fargesin, sesamin and L-asarinin, were used as marker compounds, and extraction was optimized and projected by response surface methodology (RSM) and artificial neural network (ANN). The optimal condition for ATPS with 20% *n-*propanol and 24% (NH_4_)_2_SO_4_ coupled with ultrasonic-assisted extraction including a solvent to solid ratio of 15:1, a temperature of 40 °C, and a treatment time of 55 min was obtained. Under the condition, the yield of (−)-fargesin increased 15.12%, and the purities of (−)-fargesin, sesamin and L-asarinin reached 2.222%, 1.066%, and 1.583%, with an increase of 44.38%, 25.70%, and 26.34% compared to those extracted with 95% ethanol, respectively. Coefficient of the determined (0.9855) and mean squared error (0.0018) of ANN model suggested good fitness and generalization of the ANN. Taken together, the results showed that ultrasonic-assisted ATPS can be a suitable method for extraction and enrichment of lignans from *Z. armatum*.

## 1. Introduction

Traditional extraction and separation of natural products are typically performed using organic solvents such as ethanol/water at room temperature or by heat reflux. Unfortunately, these methods are time consuming and often harmful to the environment [1]. The selective separation of target molecules can be difficult to accomplish using traditional methods because of the low concentrations of the natural small molecules in the raw material and the high costs associated with their subsequent isolation. So, the development of a new, simple method for extracting natural small molecules is required.

With the development of “green chemistry”, environmentally friendly techniques have steadily become more attractive over the last few years [2]. An aqueous two-phase system (ATPS) is one useful separation technique for the extraction and separation of a number of natural small molecules from herbs [3,4]. ATPS is a suitable alternative that integrates clarification, concentration, and partial purification in one step and is easy to scale up [5]. In the last decade, different from the ATPSs with standard batch processes, the microfluidic protocols involving ATPSs (continuous-flow processes) have been proposed, and some microfluidic devices have also been developed [6]. ATPS may be composed of short chain alcohols and inorganic salts. Generally speaking, herb powder is placed into the ATPS, and after mixing, the system becomes an emulsion. Some natural small molecules can be extracted easily with alcohol-rich droplets. Depending on the physicochemical properties of the molecules, ATPS can be used to achieve high extraction yield and purity of target compounds.

More recently, response surface methodology (RSM) has been applied for process optimization [7,8]. RSM is a collection of statistical techniques and mathematical models that are used to define the relationship between the variables and the response [9]. Artificial neural network (ANN) is often used for process modeling [10,11,12]. An ANN is characterized by a vector of input and output states, a connectivity matrix, a bias or threshold vector, a transition function, a network architecture and a learning rule. Ultimately, the ANN imitates the structure of brain function in biological neural network using mathematical modeling [13]. In each neuron, the weighted sum of the total input passes through a specific function to generate an output signal [14]. ANN has been used successfully to model the extraction of natural medicinal materials.

*Zanthoxylum armatum* DC is a very common plant having medicine and food functions throughout mainly southeast and southwest China, and cultivated in some areas of China [15]. It has been traditionally used to prevent stomach ache, tooth ache, and treat colds symptoms in the chest and abdomen [16]. Previous phytochemical investigations have revealed the presence of lignan, triterpene, amide, coumarin, alkaloid and flavonoid compounds [15]. Lignans, the characteristic constituents of *Z. armatum*, are thought to be largely responsible for anti-inflammatory activities of the plant. Now, the preparation of high purity and content of *Z. armatum* lignans for development of new anti-inflammatory drugs have become a hot topic in China.

**Figure 1:**
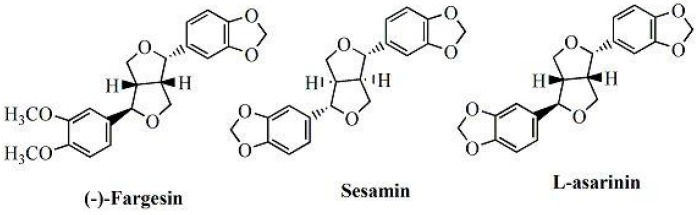
Chemical structures of three major lignans in *Zanthoxylum armatum*.

The aim of this study was to optimize the conditions for ultrasonic-assisted ATPS (UAE-ATPS) extraction of lignan compounds from *Z. armatum*. Three lignans, namely, (−)-fargesin (Far), sesamin (Ses) and L-asarinin (Asa), were used as markers to monitor extraction efficiency (Figure 1). The effects of variations in inorganic salt/alcohol type and salt/alcohol concentration on lignan recoveries were studied. RSM was used to evaluate the influence of the ultrasound temperature, extraction time and solvent to solid ratio to the lignan extraction. In addition, ANN was used to model lignan extraction parameters.

## 2. Results and Discussion

### 2.1. Phase Diagram of the Alcohol/Salts System

**Figure 2:**
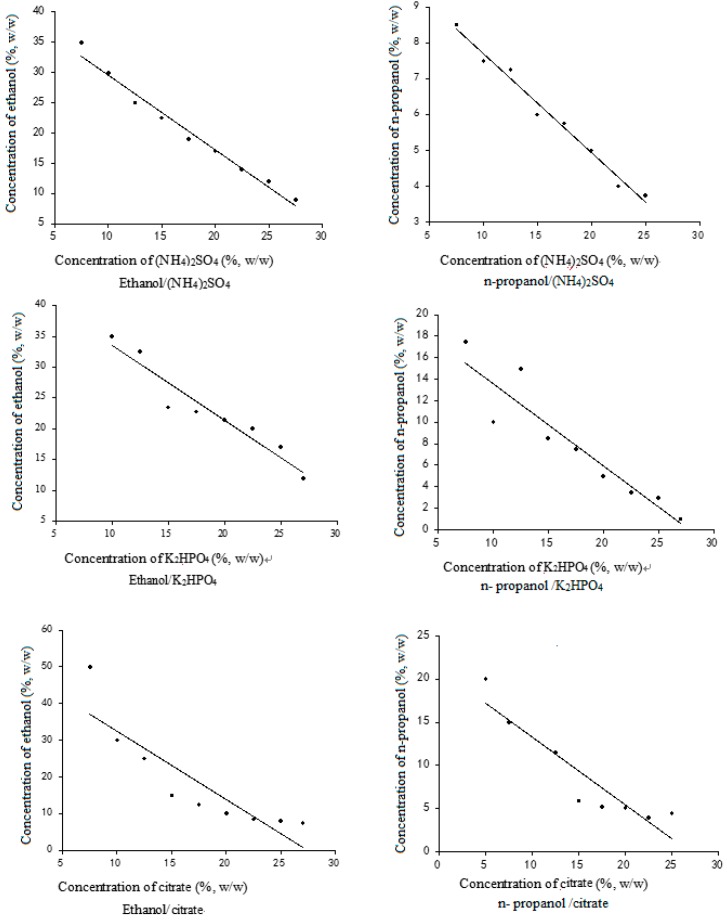
Phase diagram of different aqueous two-phase systems.

The phase diagrams of the alcohol/salt systems were determined to select an appropriate ATPS for the present study (Figure 2). Two zones separated by a curve were observed. The ethanol and K_2_HPO_4_ contents were selected from the zone above the curve. An ATPS could be easily formed above the curve. With increases in the concentration of alcohol, the curve would cause the critical point of forming ATPS, and if the concentration of salt continued to increase when at high concentration of salt level, excessive salt would be separated from the solution as solids. This phenomenon is known as solvent dilution crystallization and can be used to recover the salts after extraction [17]. Therefore, a curve must be selected for the concentrations of both alcohol and salts. Figure 2 shows that low concentrations of *n-*propanol compared to ethanol in the presence of different salts can easily form the ATPS.

### 2.2. The Selection of the ATPS System

Suitable solvents in the ATPS must be selected for the efficient extraction and enrichment of lignans. The following systems were tested for their phase-forming and partition characteristics: 24% (NH_4_)_2_SO_4_ with 20% ethanol, 24% (NH_4_)_2_SO_4_ with 20% *n-*propanol, 22% K_2_HPO_4_ with 20% ethanol, 22% K_2_HPO_4_ with 20% *n-*propanol, 24% citrate with 20% *n-*propanol, and 24% citrate with 20% ethanol. Because the system containing 24% K_2_HPO_4_ can cause the salt to separate from solution, the ATPS using K_2_HPO_4_ were consisted of 22% K_2_HPO_4_ with ethanol or *n-*propanol, instead of 24% K_2_HPO_4_.

**Figure 3:**
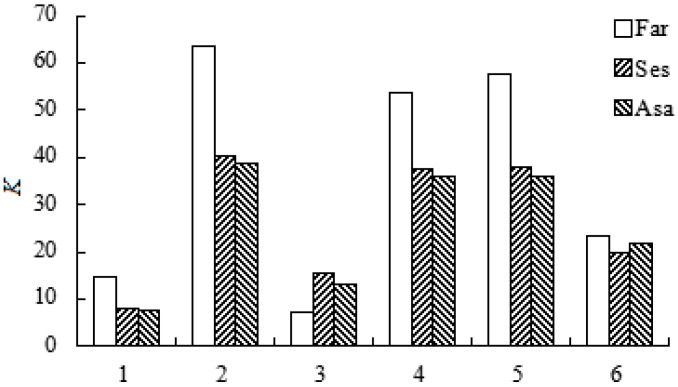
Effect of different aqueous two-phase systems on the partition coefficient (*K*) of three lignans. 1: 24% (NH_4_)_2_SO_4_/20% ethanol; 2: 24% (NH_4_)_2_SO_4_/20% *n-*propyl alcohol; 3: 22% K_2_HPO_4_/24% ethanol; 4: 22% K_2_HPO_4_/24% *n-*propyl alcohol; 5: 24% citrate/20% *n-*propyl alcohol; 6: 24% citrate/20% ethanol.

As shown in Figure 3, the ATPS with 24% (NH_4_)_2_SO_4_ with 20% *n-*propanol yielded higher *K* values than the other ATPS*.* Therefore, this ATPS was selected for further investigation. Moreover, it can be found that the partition behaviors of Ses and Asa in different ATPS were much more similar to each other than any other compound. The possible reason is that the partition behaviors are highly correlated with the polarity of the tested compounds. Even though Far and Asa have an identical absolute configuration (7*S*, 7′*R*, 8*S*, 8′*S*) while Ses has another absolute configuration (7*R*, 7′*R*, 8*S*, 8′*S*), Ses and Asa have similar partition behaviors because Ses and Asa are isomers that possess similar polarity.

### 2.3. The Effects of (NH_4_)_2_SO_4_ on the Lignan Recoveries

**Figure 4:**
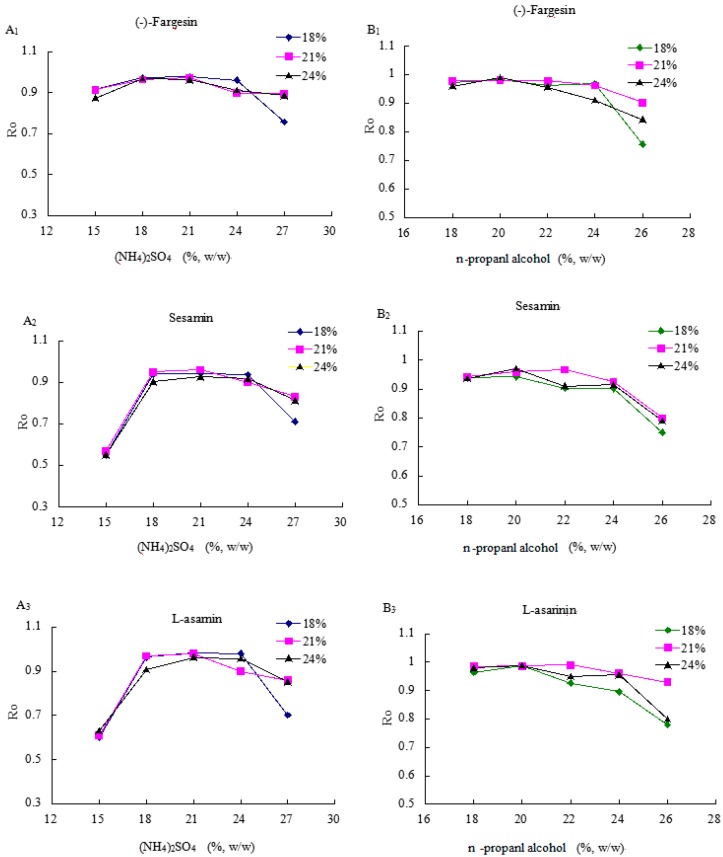
Effect of (NH_4_)_2_SO_4_ (**A**) /*n-*propanol (**B**) concentrations on the recovery (*R*_0_) of three lignans (1: Far; 2: Ses; 3: Asa)

A series of solutions containing 15%, 18%, 21%, 24% and 27% (NH_4_)_2_SO_4_ with 18%, 21% and 24% *n-*propanol were investigated to identify good ATPS with the best lignan recovery. As shown in Figure 4A_1_–A_3_, the lignan recoveries increased with increasing concentration of (NH_4_)_2_SO_4_, and the lignan recoveries became highest when the (NH_4_)_2_SO_4_ in the ATPS was 24%. The reason for this observation may be that the water would enter into the lower phase, and the concentration of *n-*propanol in the upper phase increased with the increasing salt in the ATPS. However, when the concentration of (NH_4_)_2_SO_4_ continued to increase, three lignan recoveries decreased. The reason could be that too much water entered the lower phase, and the concentration of *n-*propanol became too high, meaning that the polarity of the upper phase would be unsuitable for the three lignans. Therefore, (NH_4_)_2_SO_4_ concentrations between 18% and 24% yielded a relatively high *R*_0_, and were chosen for subsequent tests.

### 2.4. The Effects of n-Propanol on the Lignan Recovery

The ATPS consisting of *n-*propanol concentration ranging from 18%–26% with 18%, 21% and 24% (NH_4_)_2_SO_4_ were chosen to evaluate the effects of the *n-*propanol content on lignan recoveries. As shown in Figure 4B_1_–B_3_, the ATPS with 24% (NH_4_)_2_SO_4_ and 20% *n-*propanol gained the highest *R*_0_ values for three lignans, and the obtained *R*_0_ values were 0.9910 for Far, 0.9832 for Ses, and 0.9870 for Asa. It can be that the ATPS reached a suitable upper phase/lower phase ratio causing lignans to partition into the upper phase containing *n-*propanol easily. So, the optimal ATPS of 24% (NH_4_)_2_SO_4_ and 20% *n-*propanol was chosen for the subsequent test.

### 2.5. RSM Modeling

**Figure 5:**
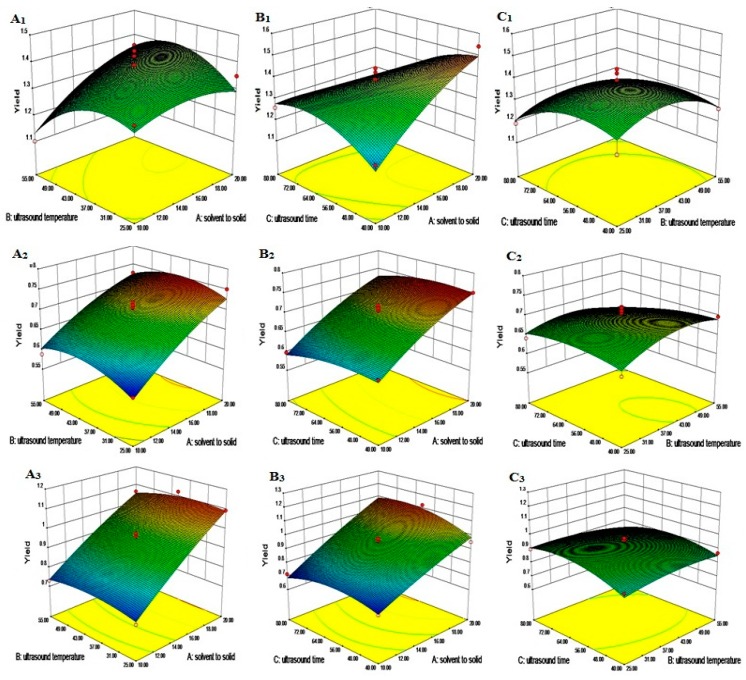
Response surface plot showing the effect of ultrasound time, temperature and solvent to solid ratio on the yield of lignans. (Figure 5X_1_, *X* = **A**–**C** )Response surface plot showing the effect of ultrasound time and solvent to solid ratio on the yield of lignans (Far (**A_1_**), Ses (**B_1_**), Asa (**C_1_**)). The time was constant at 60 min. (Figure 5X_2_) Response surface plot showing the effect of ultrasound time and solvent to solid ratio on the yield of lignans (Far (**A_2_**), Ses (**B_2_**), Asa (**C_2_**)). The temperature was constant at 40 °C. (Figure 5X_3_) Response surface plot showing the effect of ultrasound time and ultrasound temperature on the yield of lignans (Far (**A_3_**), Ses (**B_3_**), Asa (**C_3_**)). The solvent to solid ratio was constant at 15. *X* = **A**–**C**.

The application of ultrasound waves is an efficient tool to facilitate the emulsification of ATPS and to accelerate mass-transfer of liquid–liquid systems [17]. Ultrasonic time, ultrasonic temperature and solvent to solid ratio are key parameters that that contribute to enhancements in the extraction efficiency. In the study, the ATPS with 24% (NH_4_)_2_SO_4_ and 20% *n-*propanol was used to investigate the optimum conditions for UAE. The experimental yields of the three lignan markers under various experimental conditions were presented in Table 1. The effects of solvent to solid, ultrasound temperature and ultrasound time on the yields of three lignans are shown in Figure 5.

The fitted quadratic model for Far yield was given in Equation (1).

Yield = 1.39 + 0.058 × X_1_ − 0.022 × X_2_ − 0.052 × X_3_ + 0.054 × X_1_ × X_2_ − 0.11 × X_1_ × X_3_ − 0.003 × X_2_ × X_3_ − 0.039 × X_1_^2^ − 0.083 × X_2_^2^ − 0.070 × X_3_^2^ (1)

As shown in Figure 5A_1_, at a constant ultrasonic time (60 min), the yield of Far increased to a certain value with the improvement of solvent to solid ratio. This is because a higher ratio of solvent to solid implied greater concentration difference between the interior plant cells and the exterior solvent, and leaded to the increase in the driving force for the mass transfer of lignans. However, further increase in solvent to solid ratio resulted in a reversal of this trend. This might be due to the reason that a higher ratio of solvent to solid prolonged the distance of diffusion towards the interior tissues and caused a large loss during production collection [18]. The yield of Far also increased with an increasing ultrasonic temperature initially. This phenomenon is most likely due to an improvement in the mass transfer rate at higher temperatures, because at higher temperatures the solubility of the Far increased and the solvent viscosity reduced. However, with further increases in ultrasonic temperature, a decline in the yield of Far was observed.

The effects of ultrasonic time and solvent to solid ratio on the yield of Far at a constant ultrasonic temperature (40 °C) are shown in Figure 5A_2_. On the one hand, when the ultrasonic time was at a relatively low level, the yield of Far increased with increasing solvent to solid ratios. While over a relatively long period (80 min), the yield of Far increased at first, then decreased with the improvement of solvent to solid ratios. On the other hand, when the solvent to solid ratio was at a low level, the yield of Far increased initially, then slightly decreased with increasing ultrasonic time. This might be due to the time requirement of the exposure of the lignan to the release medium where the liquid penetrated into the raw materials, dissolved the lignan and subsequently diffused out from the raw materials [18]. However, a longer ultrasound extraction time may result in destruction of Far when Far in plant cells has been already sufficiently extracted. While at a high level of solvent to solid ratio, the yield of Far decreased with the increasing ultrasonic time.

A similar phenomenon in Figure 5A_1_ also appeared in Figure 5A_3_. As can be seen in Figure 5A_3_, at a constant solvent to solid ratio of 15:1, the yield of Far increased by increasing ultrasonic temperature or time, but at higher temperatures or longer ultrasonic time, the yield of Far decreased as the ultrasonic temperature or time increased.

The fitted quadratic model for Ses yield was given in Equation (2).

(2) Yield = 0.70 + 0.068 × *X*_1_ + 0.0006125 × *X*_2_ − 0.012 × *X*_3_ − 7.500E-003 × *X*_1_ × *X*_2_ + 0.001747 × *X*_1_ × *X*_3_ − 0.013 × *X*_2_ × *X*_3_ − 0.014 × *X*_1_^2^ − 0.027 × *X*_2_^2^ − 0.014 × *X*_3_^2^

In the case of Asa, the Equation (3) is as follows:

(3) Yield = 0.94 + 0.17 × *X*_1_ − 0.007125 × *X*_2_ + 0.021 × *X*_3_ − 0.012 × *X*_1_ × *X*_2_ + 0.031 × *X*_1_ × *X*_3_ − 0.021 × *X*_2_ × *X*_3_ − 0.016 × *X*_1_^2^ − 0.027 × *X*_2_^2^ − 0.061 × *X*_3_^2^

**Table 1:** Experimental design of the BBD of the true yield of lignans and the ANN predicted values.

Test Set	Extraction Conditions			Actual Yield (mg/g Sample)			ANN Predicted Values		
	Solvent to Solid (*X*_1_)	Ultrasound Temperature (*X*_2_)	Ultrasound Time (*X*_3_)	Far	Ses	Asa	Far	Ses	Asa
1	1	0	−1	1.541	0.751	0.953	1.541	0.751	0.952
2	0	0	0	1.358	0.686	0.922	1.398	0.703	0.949
3	0	0	0	1.386	0.719	0.961	1.398	0.703	0.949
4	0	1	1	1.194	0.649	0.83	1.193	0.649	0.831
5	0	0	0	1.423	0.692	0.961	1.398	0.703	0.949
6	1	1	0	1.358	0.727	1.062	1.355	0.751	0.124
7	0	−1	1	1.190	0.641	0.897	1.189	0.641	0.896
8	0	1	−1	1.259	0.697	0.868	1.259	0.697	0.869
9	0	−1	−1	1.243	0.638	0.845	1.301	0.715	0.738
10	1	0	0	1.305	0.727	1.124	1.264	0.727	1.124
11	1	−1	0	1.349	0.751	1.093	1.305	0.741	1.100
12	−1	1	0	1.100	0.588	0.729	1.126	0.583	0.704
13	0	0	0	1.423	0.706	0.977	1.398	0.703	0.949
14	−1	0	−1	1.258	0.625	0.701	1.161	0.582	0.702
15	0	0	0	1.391	0.712	0.915	1.398	0.703	0.948
16	−1	−1	0	1.309	0.582	0.711	1.314	0.582	0.711
17	−1	0	1	1.190	0.597	0.716	1.122	0.583	0.704

The complicated correlations between the yield of Ses or Asa, and the different factors studied were also observed as Figure 5B,C, respectively. Multiple regression analyses results showed that the yields of Ses and Asa are also significantly (*p* < 0.05) affected by the linear, interaction, and quadratic terms of ultrasound time, ultrasound temperature and the solvent to solid ratio. In this work, our results indicate that the solvent to solid ratio was the most important factor in determining the yields of all three lignans.

### 2.6. Modeling of ANN

**Figure 6:**
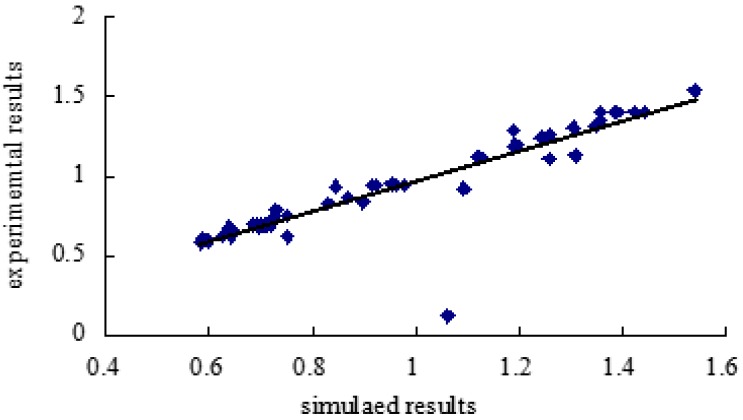
Regression plot (experimentally *vs.* predicted) using ANN model.

A feed-forward neural network trained with an error back-propagation algorithm was applied to data analysis and to model building by using the MATLAB Neural Network Toolbox. Six hidden layer networks were used for a practical feed-forward network design. After training, the coefficient determined (*R*) between the actual and predicted values was used to define the optimal number of neurons. Mean squared errors and *R* for all data sets were calculated as 0.0018 and 0.9855, respectively (Figure 6). Furthermore, the results obtained were very close to the experimental values. The results suggested that ANN models were able to successfully predict the extraction yield.

### 2.7. Comparison of ATPS and Heat Reflux Extraction

After the optimization tests were completed, a comparison of extraction using ATPS at the optimal conditions (The root and stem powders of *Z. armatum* were extracted with ATPS of 24% (NH_4_)_2_SO_4_/20% *n-*propanol according ultrasound optimum conditions: solvent to solid ratio 15:1 (*w*/*w*), 55 min, 40 °C and 250 W) and heat reflux (The powders of *Z. armatum* were extracted twice using 95% ethanol with solvent to solid ratio of 12:1 (*v*/*w*) for 1 h) was conducted. The results are shown in Table 2. The lignan yields obtained by ATPS for Far, Ses and Asa were 1.591, 0.763, and 1.133 mg/g, respectively, while the yields for traditional reflux extraction were 1.382 mg/g for Far, 0.762 mg/g for Ses and 1.125 mg/g for Asa. The Far yield using ATPS showed an apparent improvement, with an increase of 15.12% compared to by reflux extraction. On the other hand, the purities of Far, Ses and Asa using ATPS reached 2.222%, 1.066% and 1.583% with an increase in purity of 44.38%, 25.70% and 26.34%, respectively. Thus, ATPS can be a valuable alternative to conventional solvent-based extractions, resulting in both the efficient extraction and enrichment of *Z. armatum* lignans.

**Table 2:** Comparison of ATPS and conventional reflux extraction method.

Sample	Yield of Extract	Far		Ses		Asa	
	(mg/g)	Yield (mg/g)	Purity (%)	Yield (mg/g)	Purity (%)	Yield (mg/g)	Purity (%)
Extracts with ATPS	71.57 ± 1.82	1.591 ± 0.16	2.222	0.763 ± 0.07	1.066	1.133 ± 0.10	1.583
Extracts with ethanol	89.80 ± 1.33	1.382 ± 0.12	1.539	0.771 ± 0.05	0.858	1.125 ± 0.09	1.253

Data are expressed as the means ± SD (*n* = 3).

A study by Guo *et al.* [17] showed, that ATPS under optimal conditions gained an approximate yield of *Schisandra Chinensis* lignan comparable with 80% ethanol (*v*/*v*) ultrasonic extraction, but obtained high extraction purity of *Schisandra Chinensis* lignan. Similarly, it was reported that the purity of two main flavonoids (genistein and apigenin) in pigeon pea using microwave-assisted ATPS reached 0.471% and 0.233%, which was an increase of 110% and 85% compared to heat reflux extraction [1]. Combined with our present findings, it seems that ATPS is a very useful way for extraction and enrichment of natural small molecules.

## 3. Experimental Section

### 3.1. Materials

The root and stem of *Z. armatum* were collected from Nanning, Guangxi Province of China in August 2010 and identified by Professor Zhu Yi-Lin. A voucher specimen for the plant (#10021-05) was deposited in the Herbarium of School of Life and Engineering, Lanzhou University of Technology, China. The three standard lignans, namely, (−)-fargesin (Far), sesamin (Ses) and L-asarinin (Asa) were isolated and purified by our research group. All other chemicals used were of analytical-reagent grade.

### 3.2. Preparation of the Crude Lignan Extract

Dried root and stem powder of *Z. armatum* (100.0 g) were extracted twice using 95% (*v*/*v*) ethanol (2 × 1 L) with heat extraction for 1 h at 75 °C. The extract was then concentrated under reduced pressure to remove the ethanol and dried to give the crude lignan extract (about 9.0 g).

### 3.3. Preparation of Aqueous Two-Phase Systems

Three salts, namely, (NH_4_)_2_SO_4_, citrate, and K_2_HPO_4_, and two alcohols, namely, ethanol and *n-*propyl alcohol, were tested for their phase-forming abilities at room temperature. As described by Zhang *et al*. [19], the phase diagram of alcohol and salts was prepared by a turbidity titration method. A series of tubes with different amounts of salts and water were prepared. After that, different amounts of alcohol were added to each tube and mixed until the turbidity disappeared. The concentrations of alcohol and salts at different turbidity points were calculated, and a curve was plotted by alcohol and salt concentration. The amounts of added alcohol and salts were measured by weight and calculated using the following equations:

(4) $$W_{1}=m_{1}/(m_{1}+m_{2}+m_{3})$$

(5) $$W_{2}=m_{2}/(m_{1}+m_{2}+m_{3})$$

where *m*_1_, *m*_2_ and *m*_3_ respectively represent the amount of alcohol, salt and distilled water added in grams, *W*_1_ and *W*_2_ represent the mass fractions of alcohol and salt (mg/mg).

The crude lignan extract was used to evaluate several ATP systems in terms of partition capacity and recovery without the use of ultrasound. The improved method was adopted for the tests as previously described [17]. A certain concentration of (NH_4_)_2_SO_4_, citrate or K_2_HPO_4_ solution was mixed with ethanol or *n-*propanol in 15 mL graduated tubes with a conical base, respectively. Then 0.1 g of the crude lignan extract and water were added one after another to give a final mass of 10.0 g. The two phases were then separated and left to stand for 5 h after vortex mixing for 4 min. Finally, the upper and lower phases were separated using pipettes and analyzed for relative concentrations of the three lignans. The partition coefficient (*K*) and the recovery (*R*_0_) of lignans were calculated as following:

(6) $$K=\frac{C_{t}}{C_{b}}$$

(7) $$R_{0}=\frac{C_{t}V_{t}}{C_{t}V_{t}+C_{b}V_{b}}$$

where *C_t_* and *C_b_* represent the measured concentration (mg/mL) of the upper and lower phases, respectively, *V_t_* and *V_b_* represent the volumes (mL) of the upper and lower phases.

### 3.4. Ultrasonic-Assisted ATPS (UAE-ATPS) to Extract Lignans from Z. armatum

An ATPS composed of (NH_4_)_2_SO_4_ and *n-*propanol was selected based on its high upper phase recovery yield. A 100.0 g of ATPS was prepared with 1.0 g root and stem powder of *Z. armatum*. The UAE-ATPS was performed in ultrasonic cleaner (KQ-250DE, 40 kHz, 250 W, Kunshan Ultrasonic Instrument Co. Jiangsu, China). A time and temperature controller was used to change these extraction conditions. The system was filtered and left to stand for 5 h. Then, the concentrations of Far, Asa and Ses in the upper phase were measured and calculated as following:

(8) $$Yield(mg/g)=\frac{C_{t}V_{t}}{M_{0}}$$

where *C_t_* and *V_t_* represent the concentration (mg/mL) and volume (mL) of the upper phase, respectively, while *M*_0_ represent the mass of the root and stem powder used (g).

(9) $$purity=\frac{C_{t}V_{t}}{M_{1}}\times 100\%$$

where *M*_1_ represents the mass of lignan extract used (g).

### 3.5. HPLC Analysis

The HPLC system was employed to determine the contents of three lignans according to our previous report. The HPLC analysis using a liquid chromatograph system (LC-15, Shimadzu, Japan) with UV-VIS detector to analysis the concentration of Far, Asa and Ses, chromatographic separation was carried out on a Cosmosil C_18_ column(4.6 mm × 250 mm, 5 μm, Nacalai, Japan). The solvent system was composed of A (acetonitrile) and B (water), with an elution program performed as follows: 0–20 min, linear gradient from 30% A to 50% A; 20–35 min, 50% A; 35–50 min, linear gradient from 50% A to 90% A, and 50–57 min, 90% A. The total time of analysis was 57 min. The flow rate was 1.0 mL/min, the column temperature was set at 30 °C, and the injection volume was 20 μL. At 233 nm, the run times of Far, Ses and Asa were 31, 37 and 41 min, respectively. The regression lines for each compound were:

(10) *Y*_1_ = 43927*X*_a_ − 10154 (R^2^ = 0.9996)

(11) *Y*_2_ = 24760*X*_b_ − 27144 (R^2^ = 0.9996)

(12) *Y*_3_ = 28787*X*_c_ − 22359 (R^2^ = 0.9993)

*Y* represents the peak area of analyte, and *X_a_*, *X_b_* and *X_c_* represent the concentration of analyte (μg/mL), respectively.

### 3.6. RSM Design

RSM was used to optimize the UAE-ATPS conditions for the recovery of lignans. The solvent to solid ratio (*X*_1_), ultrasound temperature (*X*_2_) and ultrasound time (*X*_3_) were used as independent variables. In RSM, real values of independent variables were transformed to the coded value. Each factor in three levels were coded with values of −1, 0 and 1. While the real values were 10, 15 and 20 g/g for *X*_1_, 25, 40 and 55 °C for *X*_2_, and 40, 60 and 80 min for *X*_3_. A Box-Behnkeen design (BBD) in RSM was used to develop a response surface quadratic model for describing the extraction process.

### 3.7. ANN Modeling

ANN modeling was used to project the lignan yield. The data was analyzed in MATLAB 7 software. A three-layer feed forward back-propagation neural network with a linear transfer function was developed for modeling the extraction. The solvent to solid ratio (*X*_1_), ultrasound temperature (*X*_2_) and ultrasound time (*X*_3_) were used as independent variables, while the extraction yields of the three lignans were considered dependent variables. A network model with three neurons in the input layer and three in the output layer was adopted. The best neural network model was determined after a number of training trials. The maximum number of epochs was chosen by a trial and error approach.

## 4. Conclusions

The study represents the first report on the feasibility of ATPS extraction for *Z. armatum* lignans. The ATPS with 20% *n-*propanol and 24% (NH_4_)_2_SO_4_ coupled with ultrasound at solvent to solid ratio of 15:1, extraction time of 55 min and an extraction temperature of 40 °C was considered as optimal condition for maximizing extraction and enrichment *Z. armatum* lignans. After using UAE-ATPS, the yield of Far and purities of all three lignans were significantly improved compared to reflux extraction. These findings demonstrate that this method is very effective for the selective extraction of lignans from *Z. armatum*.
